# Cardiopulmonary Management for Severe Aortic Valve Stenosis With Noninvasive Ventilation Using a Nasopharyngeal Tube

**DOI:** 10.7759/cureus.65233

**Published:** 2024-07-23

**Authors:** Soichiro Tanimura, Kenji Morimoto, Masumi Tanaka, Osamu Saito

**Affiliations:** 1 Division of Critical Care Medicine, National Center for Child Health and Development, Setagaya, JPN; 2 Division of Critical Care Medicine, Tokyo Metropolitan Children’s Medical Center, Fuchu, JPN

**Keywords:** respiratory and circulatory support, afterload, aortic valve stenosis, noninvasive ventilation, nasopharyngeal tube

## Abstract

The nasopharyngeal tube (NT) is an effective interface for noninvasive ventilation (NIV). In cases of severe heart failure, assistance with noninvasive positive-pressure ventilation (NPPV) effectively reduces afterload and alleviates respiratory effort. We present the case of a three-day-old male neonate diagnosed with severe aortic valve stenosis (AS). In respiratory management, extubation was delayed due to increased respiratory effort and afterload, so this patient was extubated and managed with NPPV using an NT. An uncuffed endotracheal tube was inserted, initiating NIV with a positive end-expiratory pressure of 8 cmH_2_O. The patient exhibited stable vital signs post-extubation and was weaned off NPPV and transferred to the general ward. In this case of severe AS, the use of NT as an interface for NPPV demonstrated efficacy in respiratory and circulatory management. This approach could have shortened the duration of mechanical ventilation and facilitated safe postoperative care, highlighting the potential benefits of NT in managing severe heart failure.

## Introduction

Noninvasive ventilation (NIV) is an effective respiratory support for pediatric respiratory diseases, such as bronchiolitis, pneumonia, and acute respiratory distress syndrome, associated with immune disorders [1-3]. It has shown effectiveness in the postoperative management of congenital heart disease as cardiopulmonary management, and its use has recently become widespread [4].

The nasopharyngeal tube (NT) is an effective interface for NIV [5]. In cases of severe heart failure, noninvasive positive-pressure ventilation (NPPV) effectively reduces the cardiac afterload and alleviates the respiratory effort; however, studies reporting the management of neonatal cases of aortic stenosis (AS) using NPPV with NT as the interface are limited. We report a neonatal case of severe AS, which demonstrated the efficacy of NPPV used with NT for respiratory and circulatory support.

## Case presentation

Medical history

The patient was a three-day-old male infant who was born at full term after an uncomplicated pregnancy, with a birth weight of 3,335 g. The prenatal diagnosis was not performed. He had respiratory distress and cyanosis. His blood pressure was unknown, and other vital signs were heart rate of 180 beats/minute, temperature of 35.8°C, respiratory rate of 80 breaths/minute, and SpO_2_ of 90% on room air. A heart murmur was immediately detected, and cardiac ultrasound findings led to a diagnosis of severe AS. Initially, the pressure gradient between the left ventricle and aorta was ≥80 mmHg. The patient was deemed suitable for cardiac catheterization therapy and was transferred to our hospital.

Cardiac catheterization treatment

On cardiac catheterization, the left ventricular pressure was 180 mmHg, and the aortic pressure was 50 mmHg. The resulting pressure gradient was approximately 130 mmHg. Balloon dilation was performed twice, reducing the pressure gradient to approximately 50 mmHg under general anesthesia The first dilation was performed using a balloon dilatation catheter with a 5-mm diameter, corresponding to 90% of the valve’s annular diameter, and the second dilation was performed using a balloon dilatation catheter with a 6-mm diameter, corresponding to 100% of the annular diameter. After the procedures, the patient was admitted to the pediatric intensive care unit (PICU) under mechanical ventilation and administered adrenaline and dobutamine.

Pediatric intensive care unit management

The initial vital signs upon admission were blood pressure of 50/30 mmHg in the upper limbs, heart rate of 182 beats/minute, temperature of 36.0°C, and SpO_2_ of 98% on oxygen supplementation at an FIO_2_ of 0.30. After admission, deep sedation and muscle relaxation management were performed. The latter was continued until postoperative day two. Postoperative myocardial contractility was negative on echocardiography. Adrenaline tapering began on postoperative day one and was discontinued on postoperative day two. Noradrenaline was administered to maintain the diastolic blood pressure and was discontinued on postoperative day three. Dobutamine tapering began on postoperative day five and was discontinued on postoperative day eight. Respiratory management was performed using the synchronized intermittent mandatory ventilation mode with a peak inspiratory pressure (PIP) of 22-24 cmH_2_O and positive end-expiratory pressure (PEEP) of 5 cmH_2_O for assistance. From postoperative day four, respiratory management primarily focused on spontaneous breathing and ventilator assistance was reduced to PIP of 18 cmH_2_O on postoperative day nine. Subsequently, daily spontaneous breathing trials were implemented; however, the heart rate and respiratory rate increased, and the respiratory effort intensified, leading to the decision to delay extubation. An increased afterload caused by reduced ventilator assistance was suspected of being one of the possible causes of this complication.

Nasopharyngeal tube utilization

**Figure 1:**
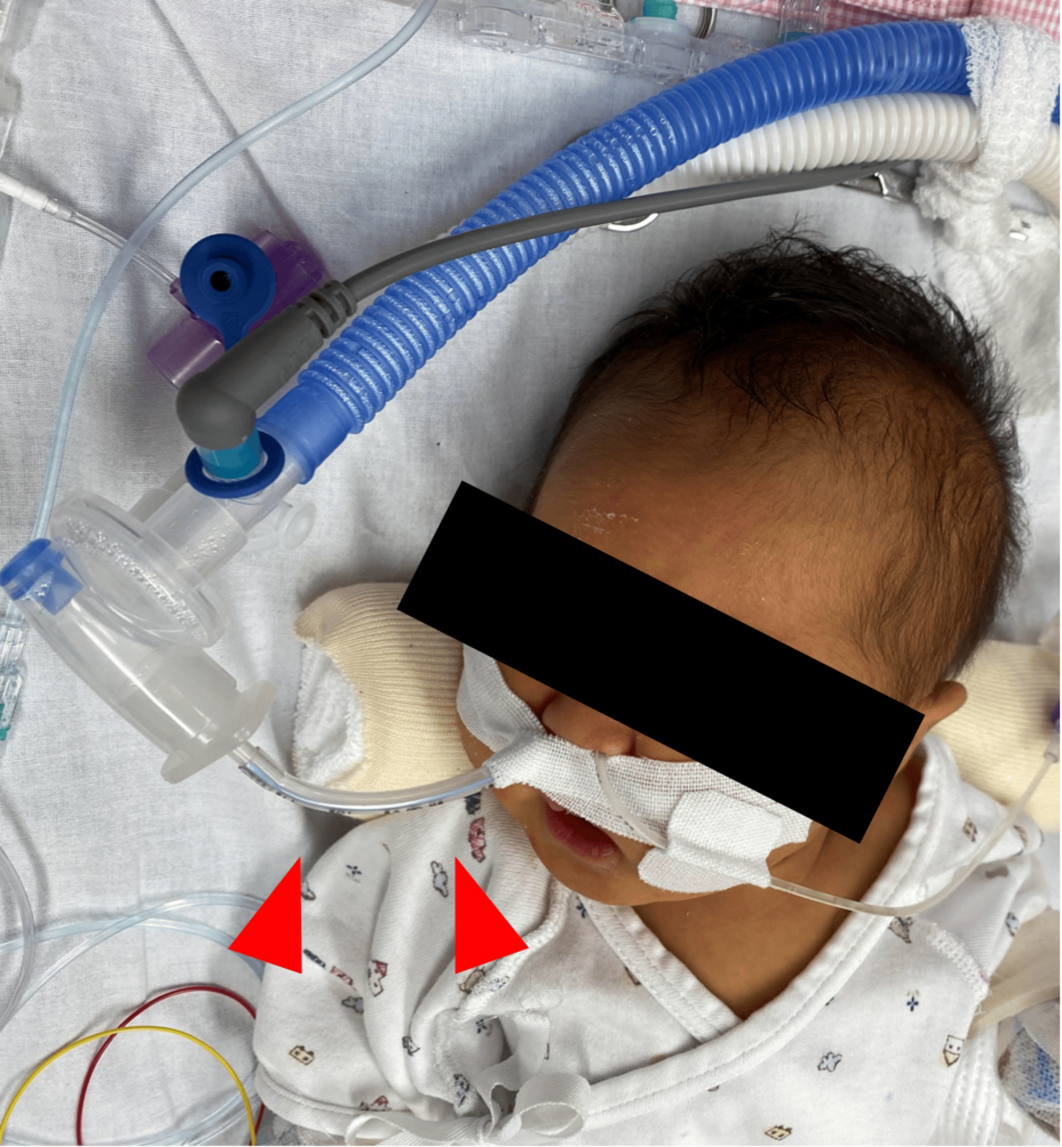
The nasopharyngeal tube used as an interface for noninvasive ventilation.

Given the patient’s condition, NPPV with an NT was used and the patient was extubated on postoperative day 12. A Shiley™ 3.0-mm uncuffed endotracheal tube of 12 cm length was inserted 6 cm into the right nostril (Figure 1). Respiratory management was initiated on the NIV mode with a PEEP of 8 cmH_2_O. Vital signs did not deteriorate post-extubation, and no concerns were noted about the child’s acceptance even without sedation. Ventilator assistance was gradually reduced to a PEEP of 5 cmH_2_O on postoperative day 13. The NPPV was eventually discontinued, and the patient was transferred to the general ward on postoperative day 14 with no complications.

## Discussion

We experienced a case of NPPV use with an NT that might have shortened the duration of invasive mechanical ventilation and allowed for safe respiratory and circulatory management in a neonate with severe AS.

In conditions such as severe AS, which can lead to left heart failure and an increased afterload associated with left ventricular outflow tract obstruction (LVOT), reducing the afterload has favorable effects on the cardiovascular system [6]. The left ventricular afterload can be calculated using the following formula: LV afterload is proportional to the difference between left ventricle intracavitary and intrapleural pressure. An increase in the intrathoracic pressure reduces the afterload and increases the cardiac output, as alleviating heart failure has already been reported [1,7]. A higher post-extubation intrathoracic pressure was advantageous for circulatory management; therefore, NPPV was used for respiratory and circulatory support.

Compared with the other interfaces of NPPV, NT is readily available and inexpensive and has the additional benefit of reducing the nursing burden by resolving leaks exacerbated by head movements or positional changes [5,8,9]. By contrast, NT has several drawbacks. First, the risk of epistaxis during tube insertion is not negligible, particularly in patients receiving an anticoagulant [10]. Second, confirming the tube’s position is crucial in maintaining PIP and PEEP and avoiding overlooking esophageal intubation [11]. Third, regular tube suctioning is required to prevent tube occlusion [12]. Fourth, it is necessary to secure the NT in the correct position to prevent esophageal intubation [13].

The use of NIV for respiratory management after cardiac surgery has been increasing, and studies have reported its utility in avoiding invasive mechanical ventilation [4,5,14]. Furthermore, studies comparing patients with elective, post-extubation, and initial NPPV have demonstrated that the former had a shorter intensive care unit stay [15]. This case report highlights the novelty of NPPV with an NT in managing an LVOT obstruction and demonstrates its potential to alleviate afterload and facilitate respiratory and circulatory management. The use of NT as an interface for NPPV provides a more straightforward, less invasive respiratory and circulatory management strategy for severe heart failure.

## Conclusions

We reported a case of using NIV with NT in a newborn with severe AS for respiratory and circulatory management of heart failure. While NT is recognized as a useful interface widely used in NIV, its proactive utilization in heart failure management remains limited. Particularly in neonates, there is scarce reporting on the use of NT-assisted NIV for acute AS management. In this case, NIV with NT potentially facilitated earlier extubation and reduced cardiac afterload. The findings demonstrate the utility of the NT in the cardiorespiratory management of severe heart failure including AS.
